# Roles of anesthetics in modulating neutrophil extracellular traps formation: a review of current literature

**DOI:** 10.1097/MS9.0000000000004785

**Published:** 2026-02-16

**Authors:** Mohmad H. Alsabani

**Affiliations:** a Anesthesia Technology Department, College of Applied Medical Sciences, King Saud bin Abdulaziz University for Health Sciences, Riyadh, Saudi Arabia; b King Abdullah International Medical Research Centre, Riyadh, Saudi Arabia

**Keywords:** anesthesia, inhalational anesthetics, intravenous anesthetics, neutrophil extracellular traps, perioperative, surgery

## Abstract

Neutrophil extracellular traps (NETs) represent a critical defensive mechanism against pathogens. However, dysregulated NETs have been implicated in a range of pathological conditions. More recently, increasing attention has been given to its implications in perioperative settings. This has led to increasing interest in how common anesthetics would modulate the neutrophil ability to form NETs. Although their ability to modulate the immune system has long been recognized, a better understanding of the impact of common anesthetics on NETs release is still needed. This may reveal considerations for optimizing immune function and personalizing anesthetic techniques, especially for vulnerable perioperative and intensive care unit patients. In this review, we comprehensively discuss recent advancements in understanding the role of common anesthetics in modulating the capacity of neutrophils to form NETs and the related mechanisms. Gaining insights into the effects of anesthetics on NETs may guide new strategies to reduce complications and improve the recovery and survival of perioperative and ICU patients.

## Introduction

Neutrophils are central mediators of the innate immune system. These cells are among the primary cells that fight against infection. As “frontline” cells in host defense, neutrophils use a variety of mechanisms to protect the host against invading pathogens via well-characterized killing mechanisms, including phagocytosis, degranulation, and reactive oxygen species (ROS) generation^[1]^. In 2004, an entirely new antimicrobial mechanism of neutrophils has been uncovered upon encountering bacteria, which was named neutrophil extracellular traps (NETs)^[2]^. NETs involve the extracellular release of decondensed DNA, forming a network-like structure that captures and kills pathogens via proteins and peptides derived from neutrophil granules. Nicotinamide adenine dinucleotide phosphate (NADPH) oxidase tightly links the release of NETs to the production of ROS^[3]^. This was confirmed when patients with deficiencies in the NADPH oxidase enzyme were unable to produce NETs and suffered frequent infections^[3,4]^. The positive role of NETs is to capture and prevent the spread of pathogens. However, NETs also negatively impact the progression of numerous diseases. For example, NETs are linked to the initiation and propagation of thrombosis, which has also been linked to the development of multiple organ dysfunction (MOD)^[5–7]^.

**HIGHLIGHTS**

- Neutrophils exhibit dual roles in perioperative period, with neutrophil extracellular traps (NETs) influencing both surgical trauma recovery and wound healing processes.
- Common anesthetics can alter neutrophil ability to form NETs, and its efficacy vary by drug type, dose, and patient case context.
- The lipid carrier of propofol inhibits the NETs-forming capacity of neutrophils *in vitro*, and its clinical effects remain minimal and inconclusive.
- Local anesthetics generally reduces NETosis *in vitro* at clinically relevant doses and in patients with cancer, while dexmedetomidine reduces NETs-associated biomarkers in cancer patients.
- Additional preclinical and clinical investigations are necessary to understand how anesthetics influence NET formation, which could guide anesthetic choice and dosing to optimize perioperative immune function, particularly in vulnerable or immunocompromised patients.

Since the discovery of NETs, numerous studies have evaluated how different physiological, pathological, and biochemical factors can either drive or inhibit NETs, thereby enhancing our understanding of NET-mediating pathways and their role in disease progression and patient outcomes^[8,9]^. Surgical and intensive care unit (ICU) patients widely use anesthetic agents. Proper innate immune function is essential for the optimal postoperative recovery of surgical patients and ICU patients who are at significant risk of opportunistic and nosocomial infections. Previous preclinical and clinical investigations have characterized the roles of most anesthetic agents and other perioperative-related medications in modulating immune functions, including neutrophils^[10]^. The interaction between anesthetic agents and neutrophil functions is complex and may produce clinically significant effects. For example, anesthetic agents that inhibit neutrophil function may alter immune function in a manner that could harm the host and subsequently produce undesirable outcomes, such as postoperative infection or delayed wound healing^[11]^. On the other hand, anesthetic agents that reduce immune response or suppress neutrophil function may produce desirable effects, such as reducing the severity of ischemia-reperfusion injury (IRI)^[12,13]^.

Major procedures, such as cardiac^[14]^, orthopedic and trauma^[15]^, and cancer surgeries^[16]^, are consistently associated with elevated levels of circulating NETs postoperatively, likely triggered by extrinsic mechanical stimuli (e.g., cardiopulmonary bypass) and operative trauma^[14]^. The interplay between surgical stress and anesthetics administration may contribute to immunosuppression, with certain anesthetic techniques demonstrating the capacity to attenuate the release of NETs. For example, thoracic-epidural anesthesia, when compared to general anesthesia, has been reported to blunt NETs marker 24 hours after laparoscopic resection for colorectal cancer^[17]^. However, a clear gap in knowledge still exists regarding the dynamics of NETs markers during perioperative stages and whether the current evidence regarding the influence of anesthetics on NETs will translate into clinically meaningful long-term outcomes, especially for high-risk surgical patients. To date and despite growing interest in NETs, few comprehensive reviews have synthesized the emerging evidence of NETs involvement in perioperative patients or contextualized the expanding literature on the anesthetic modulation of NETs. This review aims to fill this gap by providing a comprehensive evaluation of the roles of NETs during the perioperative period and the profound effects of common anesthetic agents on neutrophils, particularly NET formation. We also outline the mechanisms through which anesthetic agents impact NETs formation. This study was conducted in accordance with the transparency in the reporting of artificial intelligence (TITAN) guideline^[18]^.

## Methods

An extensive literature search was conducted across several academic databases, including PubMed, Google Scholar, and Scopus, with the aim of identifying relevant articles examining how common anesthetic agents impact neutrophil capacity and mechanisms for generating NETs. The search employed a range of keyword combinations such as “neutrophil extracellular traps,” “NETs,” “anesthetic agents,” “surgical stress” “intravenous anesthetics,” “inhalational anesthetics,” “local anesthetics,” “ROS production,” and “Immune modulation,” The inclusion criteria for the selected studies focused on NETs in the perioperative patients and their roles in the context of anesthetic management. No restrictions were placed on the study design and only studies published in English were considered. Data were extracted from the selected studies and carefully reviewed, with methodology and findings critically evaluated.

## Pathways associated with NETosis

In 1996, researchers reported that neutrophils undergo cell death, which is different from both necrosis and apoptosis, after being chemically stimulated with phorbol 12-myristate 13-acetate (PMA)^[19]^. This type of cell death occurs in a series of steps, starting with loosening of the chromatin structure, followed by enlargement of the nucleus, the leakage of the nucleus’s contents into the cytoplasm, and ultimately the disruption of the cell membrane. The significance of this observation was not acknowledged until the Zychlinsky laboratory reported that PMA-induced cell death led to the release of a unique host defense structure called NETs^[2]^. In an *in vitro* system, PMA or interleukin-8 (IL-8) induced the release of DNA structures covered with histones and elastase, which resembled web-like structures.

### Suicidal NETosis

To date, researchers have identified two distinct forms of NETs^[20]^. The initial form involves the activation of protein kinase C (PKC) via the rapidly accelerated fibrosarcoma/mitogen-activated protein kinase kinase/extracellular signal-regulated kinase (RAF–MEK–ERK) signaling cascade, which leads to the stimulation of NADPH oxidase^[3,21]^. This pathway results in increased levels of ROS, leading to the degradation of nuclear, granular, and cytoplasmic membranes. Neutrophil elastase (NE) also translocates into the nucleus, resulting in histone cleavage^[22]^. This process allows for chromatin decondensation and the subsequent release of NETs. NETs are extracellular structures composed of decondensed DNA decorated with many antimicrobial proteins and peptides, such as histones, elastases, myeloperoxidases (MPO), and metalloproteinases (MMPs). MPO plays an essential role in chromatin breakdown and nuclear envelope design^[22]^. This type of NET formation, known as suicidal NETosis, was first described by Steinberg and Grinstein, who introduced the term to characterize this specific form of neutrophil death^[23]^.

### Vital NETosis

The second form of NETs release is known as vital NETosis, in which neutrophils, upon stimulation by microbial experience or lipopolysaccharides (LPS), release NETs but remain active and functional^[24–26]^. This pathway involves pathogen-specific activation pathways, such as Toll-like receptors (TLRs). This leads to the activation of peptidyl arginine deiminase-4 (PAD-4), a protein that is responsible for the hypercitrullination of nuclear histones^[27,28]^. This process involves the breakdown of the nuclear membrane and the formation of vehicles, resulting in the release of NETs^[24,29]^. Importantly, this does not damage the cytoplasmic membranes, thus maintaining the structural integrity of the neutrophils.

## NETs during the perioperative period

NETs are being recognized as critical components of the immune system, demonstrating diverse roles beyond their typical ability to trap pathogens. NETs combat infections by trapping, immobilizing, and killing gram-negative and gram-positive bacteria^[30]^, viruses^[31,32]^, fungi^[33]^, and parasites^[34,35]^. Consequently, they prevent the spread of invading microorganisms and defend the host. While the mechanisms and implications of NETs have been investigated in various clinical conditions, emerging evidence suggests that NETs are also recognized in surgical settings^[15,36–38]^. This is because of the complex involvement of neutrophils in host protection, tissue injury, immunological responses, and physiological stress^[39–41]^. Gaining a comprehensive understanding of the consequences of NETs throughout the surgical phase is therefore crucial for enhancing patient care and surgical outcomes.

### Surgical trauma and NETs

Surgical trauma triggers complex physiological responses involving the endocrine, metabolic, hemodynamic, and immune systems, which can persist for several days^[42]^. The immune response triggered by surgical trauma involves the activation of both innate and adaptive immune cells. Neutrophils, the initial responders to tissue damage, quickly migrate to the surgical site and regulate early inflammatory processes. These processes eliminate pathogens, minimize tissue damage, clear dead cell residues, and promote healing processes^[43]^. The impact of surgical stress on NETs is commonly studied in the context of sterile inflammatory injuries, particularly IRI. Preclinical studies suggest that excessive NET release occurs in various IRI models of different organs^[44]^. For example, neutrophils play a critical amplifying role during the liver IRI response, and excessive NET formation is observed in both liver tissue and serum during IRI. Damaged hepatocytes release damage-associated molecular patterns (DAMPs), such as high mobility group box 1 (HMGB1) and histones, which stimulate NETs release. The attenuation of NETs via the use of DNase I and PAD4 inhibitors reduces inflammation^[45]^. More recently, a pilot clinical study investigated how the severity of surgical trauma influences NETs formation^[46]^. This study showed that severe surgical trauma caused by aortocoronary bypass results in an impaired ability of neutrophils ability to form NETs compared with mild surgical trauma caused by transcatheter/percutaneous aortic valve implantation. However, it is not clear whether this compromised function of neutrophils can contribute to poor postoperative recovery due to adverse postoperative outcomes such as infection.

### Wound healing and NETs

The role of neutrophils extends beyond responding to surgical trauma; they also play a significant role in the postoperative wound healing process. However, their roles are double-edged and may range from the normal postoperative wound healing process to extended inflammatory responses that trigger postoperative sepsis and neoplastic dissemination^[36,47]^. Wound healing is a multiphase process that is influenced by a wide range of factors, such as infection, diabetes mellitus, obesity, and sepsis. Soon after tissue damage, a significant contribution from the coagulation, inflammatory, and immune systems occurs during the wound healing process to prevent excessive fluid and blood loss and infection^[48]^. NET-associated components have been reported to participate in wound healing. Granule-derived components of NETs contribute to the normal wound healing process, but when these components are overexpressed, they can be harmful. For example, fluid obtained from chronic wounds has excessive expression of NE, and inhibiting this enzyme was linked to a faster wound healing process by re-epithelialization^[49]^. NE can impede wound healing through many mechanisms, including breakdown of the wound matrix^[50–52]^ and proteolytic cleavage of substances, such as platelet-derived growth factor or vascular endothelial growth factor, which are essential components of the natural healing process^[51]^. Furthermore, NE plays a role in converting proepithelin, a different growth factor, into epithelin. The main purpose of epithelin is to attract and activate neutrophils, which may lead to an overly inflammatory environment in wounds that do not heal properly^[53]^. Consequently, these findings directly link NE to the underlying mechanisms involved in impaired wound healing.

## Influence of anesthetics on NETs

Surgery triggers extended physiological responses across the endocrine, metabolic, hemodynamic, and immune systems. Although anesthetic agents typically have minimal impact on immune function in healthy individuals, their influence can become significant in immunocompromised patients with cancer. Certain anesthetics can alter perioperative immune function and subsequently influence postoperative outcomes^[54,55]^. In addition, surgery-induced NETs have been reported to enhance the survival of tumor cells, promote metastatic progression, and disrupt antitumor immune responses^[56]^. Recent advances in perioperative oncology outlines the possible interplay between NETs and effectiveness of immunotherapies, which now play a central role in cancer care. Emerging evidence indicates that targeting NETs in colorectal cancer demonstrated a promising strategy to improve immunotherapy effectiveness^[57]^. Recently, large-scale informatics analysis demonstrated rapidly expanding research studies in neoadjuvant immunotherapy, with particular focus on the efficacy and safety of neoadjuvant immunotherapy^[58]^. Machine learning investigation on immunotherapy-related adverse events further underscores the importance of understanding perioperative immune toxicity and developing strategies to predict and manage these risks to ensure safe and effective neoadjuvant immunotherapy treatment^[59]^. Collectively, these insights suggest that modulating NETs formation through anesthetic choice or technique may represent an additional strategy for cancer patient that may help to balance perioperative immune function, reduce postoperative tumor metastasis, and ultimately ensure more effective neoadjuvant immunotherapy. Table 1 summarizes commonly used anesthetic agents and their reported effects on NETs formation.

**Table 1:** Summary of anesthetic agents and their reported outcomes on NETs formation

Anesthetic agent	Reported effect on NETs	Mechanisms/Notes	Study settings	References
Propofol	Mixed/ Potentially inhibitory	Inhibits NETs via lipid emulsion; reduces ROS, p-ERK, and HOCl; slight effect from pure propofol; insignificant NETs reduction in clinical study (breast cancer surgery)	In vitro (PMA-stimulated neutrophils); Clinical study (breast cancer surgery)	^[60–63]^
Dexmedetomidine	Mixed/Potentially Inhibitory	Hypothesized to inhibit NETs via HMGB1/TLR and RAGE pathways; clinical evidence supports reduction in NETs biomarkers (MPO and H3Cit) in NSCLC patients	In vitro (MRSA/PMA stimulated neutrophils); Clinical study (NSCLC patients)	^[64–66]^
Sevoflurane (volatile)	No significant effect	No significant change in MPO or H3Cit after surgery	Clinical study in breast cancer patients	^[67]^
Lidocaine (local)	Dose- and context-dependent; generally inhibitory at clinical doses	Inhibits NETs via suppression of HMGB1 and G-CSF; delays the onset of NETosis at clinically relevant concentrations; higher doses accelerate NETosis; clinical evidence supports reduction in NETs biomarkers (MPO and H3Cit) in NSCLC patients	In vitro, Clinical study (NSCLC patients)	^[68–70]^
Ropivacaine (local)	Variable/Dose-dependent	Clinically relevant doses of Ropivacaine significantly reduced NETosis in PMA-stimulated neutrophils	In vitro	^[68]^
Bupivacaine	Variable/Dose-dependent	Higher concentrations accelerate NETosis; lower doses delay onset	In vitro	^[69,70]^
Levobupivacaine	Variable/Dose-dependent	Low doses delayed NETosis while higher doses accelerated it	In vitro	^[70]^
Opioids	Unknown	Known to modulate neutrophil function, but no direct studies on NETosis	No data on NETs	-

NETs, neutrophil extracellular traps; ROS, reactive oxygen species; p- ERK, phosphorylated extracellular signal-regulated kinase; HOCL, hypochlorous acid; PMA, phorbol 12-myristate 13-acetate; MRSA, methicillin-resistant *Staphylococcus aureus*, HMGB1, high-mobility group box1; TLR, toll like receptor; RAGE, receptor for advanced glycation end products; MPO, myeloperoxidases; H3Cit, citrullinated Histone H3; NSCLC, non-small cell lung cancer.

### Propofol

Propofol (2,6-diisopropylphenol) is the most frequently used hypnotic agent for induction of anesthesia because of its rapid onset and minimal side effects. In addition, propofol is used for the maintenance of anesthesia during surgery and for ICU patients requiring long-term sedation^[71]^. Propofol is an alkylphenol derivative that is formulated in a lipid emulsion and, therefore, is insoluble in aqueous solutions^[72]^. It is believed that propofol acts by directly opening the chloride channel of the gamma-aminobutyric acid (GABA) type A receptor^[73]^. However, it is worth mentioning that neutrophils do not express the GABAA receptors^[74,75]^.

Meier and colleagues demonstrated, with clinically relevant plasma concentrations of propofol, that the production of ROS and NETs release were reduced^[60]^. Propofol lipid emulsion and lipid emulsion alone produced comparable abilities to decrease NETs release. Neither agent hinders the ability of human neutrophils to effectively kill methicillin-resistant *Staphylococcus aureus* (MRSA). In addition, neither propofol nor lipid emulsion were impacted by bacterial growth. Similar findings that propofol lipid emulsion attenuated NET formation and ROS production in the presence of PMA were also shown in another study by Chen and colleagues^[61]^. These inhibitory effects occurred with clinically relevant plasma concentrations of propofol lipid emulsion and were mediated by reduced extracellular regulated kinase phosphorylation (p-ERK) and hypochlorous acid (HOCl) production. In another study, Chen and colleagues confirmed that the NET-inhibiting capacity of propofol was attributed to its lipid carrier, with a slight contribution from propofol^[62]^. They compared the NETs release of propofol lipid emulsion, lipid emulsion alone, and pure propofol. The greatest NET inhibitory effect was observed after treatment with either the propofol lipid emulsion or the lipid emulsion alone. However, pure propofol had a slight inhibitory effect on PMA-induced NETs compared with propofol lipid emulsion and lipid emulsion alone. Reductions in p-ERK and HOCl were the major pathways mediating the inhibitory effects of propofol lipid emulsion and lipid emulsion on NETs.

Clinically, in the context of cancer resection surgery, the use of intravenous propofol alone as the sole anesthetic technique results in a minor and insignificant decrease in H3Cit serum concentrations from the preoperative stag to the postoperative stage^[63]^.

The current lack of human studies and inconclusive findings from all studies suggest that the lipid carrier propofol plays an essential role in blocking NETosis. This warrants further prospective mechanistic studies and large randomized controlled trials (RCTs) to fully comprehend the effect of propofol on NET formation and its implications for the long- and short-term outcomes of surgical and critically ill patients.

### Dexmedetomidine

Dexmedetomidine, an α2-adrenoreceptor, is an anesthetic drug that is becoming more important in surgical and ICU settings because of its pain-relieving and sedative effects^[76]^. Unlike the receptor-binding site of propofol, neutrophils express adrenergic receptors, including the α2-adrenoreceptor. In addition to its clinical value as a sedative, dexmedetomidine has been shown to play an anti-inflammatory role and affect various immune cells, including neutrophils. Previous evidence suggests that dexmedetomidine inhibits oxidative bursts but not the production of superoxide anions or the phagocytosis of neutrophils^[77–79]^.

Preclinical studies conducted on sedatives have demonstrated that, compared to GABA agonists like benzodiazepines and propofol, dexmedetomidine has superior anti-inflammatory and bacterial clearance properties^[80–82]^. Additionally, administering dexmedetomidine reduces the levels of IL-6 and TNF-α in animals with severe inflammation^[83,84]^, as well as the levels of IL-1, IL-6, TNF-α, and CRP in humans following surgery^[85,86]^.

Studies on the effects of dexmedetomidine on the NET-forming capacity of human neutrophils are limited. In a recent hypothesis, Jane *et al* proposed that dexmedetomidine may have the potential to inhibit NETs in coronavirus disease (COVID-19) patients^[64]^. The authors explained a detailed molecular mechanism through which dexmedetomidine might inhibit NETs. In summary, the activation of the nod-like receptor family pyrin domain-containing protein 3 (NLRP3) inflammasome and the production of DAMPs, such as HMGB1, are key outcomes of severe acute respiratory syndrome coronavirus 2 (SARS-CoV-2) infection. HMGB1 binds to various receptors, including receptor for advanced glycation end products (RAGE) and TLR-4. Both the HMGB1/RAGE and HMGB1/TLR pathways have been shown to mediate NETs.^[87,88]^. In their hypothesis, the authors predicted that dexmedetomidine may inhibit HMGB1/TLR and HMGB1/RAGE-mediated NET release^[64]^.

The influence of dexmedetomidine on isolated human neutrophils was studied previously in the presence of MRSA or PMA to induce NETosis^[65]^. In the presence of either activating factor, dexmedetomidine did not impact the formation of NETs at therapeutically relevant concentrations or even at supratherapeutic concentrations. In a rat model of sepsis-related renal injury, dexmedetomidine attenuated proinflammatory cytokines and NE activities and improved renal function^[89]^.

Clinically, dexmedetomidine reduces postoperative NETs, including H3Cit and MPO, and biomarkers of tumor metastasis in the context of video-assisted thoracic surgery (VATS) in patients diagnosed with non-small cell lung cancer (NSCLC). A potential mechanism could involve the stimulation of α2-adrenergic receptors on neutrophils by dexmedetomidine, leading to neutrophil desensitization to cytokine activation^[66]^. Cytokines regulate neutrophil activity and contribute to the generation of NETs by activating NADPH oxidase and MPO^[66,90,91]^. Of course, the current clinical findings are promising, despite conflicting preclinical data, and suggest a potential inhibitory role in NETs and cancer-related biomarkers. Further clinical randomized controlled studies are still necessary. In particular, it would be of great interest to elucidate how such perioperative modulation of NETs might influence the long-term outcomes of these patients, most importantly tumor metastasis and recurrence.

### Inhalational anesthetic agents

Inhalational anesthetics, excluding nitrous oxide and xenon, are either halogenated alkanes (such as halothane) or halogenated ethers (such as isoflurane, enflurane, sevoflurane, and desflurane). They are commonly used in surgical patients to induce hypnosis, amnesia, and immobility in the context of noxious stimuli. Volatile anesthetics affect neutrophil function by reducing ROS generation, chemotaxis, and adherence to endothelial cells^[92–94]^. This inhibition of neutrophil activity may be helpful, particularly in ischemic conditions, by reducing the harmful consequences of excessive neutrophil activation^[95]^. *In vivo* investigations have yielded comparable results, with volatile anesthetics such as isoflurane demonstrating protective effects in an animal model of LPS-induced lung injury^[96]^.

To date, one recent pilot randomized clinical trial has evaluated the effects of different anesthetic techniques on the expression of NETs-associated biomarkers following breast cancer resection. In this study, sevoflurane did not significantly influence the postoperative levels of NETs associated biomarkers, including MPO and H3Cit^[67]^.

### Local anesthetic agents

The use of local anesthetics is essential for modern anesthesiologic procedures, including epidural anesthesia, spinal anesthesia, and local nerve and wound infiltration. Local anesthetics are also commonly used to prevent pain, especially in perioperative and cancer care settings. They predominantly impact neurons by inhibiting sodium ion influx. They also retain some immunomodulatory effects on neutrophils. Although they are generally considered electrically non-excitable, the primary mechanisms for their modulations are believed to involve the inactivation of voltage-gated sodium channels, reducing vascular adhesion or blocking inflammatory mediators, and the ability to migrate across endothelial cells^[97,98]^.

Local anesthetics have been investigated as therapeutic agents for inflammatory disorders. Lidocaine has been shown to protect cells against inflammation by decreasing neutrophil adherence and inhibiting the generation of superoxide anions ^[36,37]^. *In vitro*, lidocaine has been shown to inhibit the release of the inflammatory mediator IL-1B. Lidocaine and bupivacaine, along with a few other local anesthetics, can limit the production of ROS and inhibit granulocyte adhesion, phagocytosis, and migration. Notably, the impact of local anesthetics on neutrophil function has been primarily observed at concentrations that are unlikely to be reached during clinical use^[99,100]^.

The effects of local anesthetics on NETosis have been reported *in vitro* in resting and PMA-stimulated neutrophils^[68]^. This study aimed to compare the effects of ropivacaine and lidocaine at concentrations relevant to clinical practice. The effects of lidocaine and ropivacaine on NETosis may vary depending on the activation state of neutrophils (resting vs. PMA-stimulated) and the concentration of the drugs used. At clinically relevant concentrations, neither lidocaine nor ropivacaine induced NETosis. Although lidocaine had a minimal stimulatory effect on NETosis in PMA-stimulated neutrophils at relatively high concentrations, ropivacaine appeared to reduce NETosis in resting neutrophils at concentrations that are clinically relevant. On the other hand, Kolle *et al* reported that bupivacaine and lidocaine resulted in a dose-dependent effects on NETosis in neutrophils^[69]^. Lidocaine demonstrated biphasic responses, with low drug concentrations demonstrating delayed NETosis and higher concentrations accelerating NETosis. Additionally, Sixt and colleagues^[70]^ demonstrated that the use of clinically relevant concentrations of bupivacaine, levobupivacaine, lidocaine, and ropivacaine can inhibit neutrophil chemotaxis and the production of ROS. The authors showed that the impact of local anesthetics on NETs varies and is dose dependent. Compared with both the control and low concentrations of bupivacaine, higher concentrations of bupivacaine resulted in a significant decrease in the half-maximal time to NETosis. Levobupivacaine slightly delayed NETosis or caused no major change at lower concentrations and slightly accelerated NETosis at higher concentrations compared to control. Lidocaine exhibited a comparable trend, with a slight delay in NETosis at lower concentrations and a notable accelerating effect on NETosis onset at higher doses. Their findings revealed that lidocaine and bupivacaine induce NETosis at an earlier stage, whereas ropivacaine and levobupivacaine slightly accelerate the time to half-maximal NETosis compared with PMNs without local anesthetics.

Taken together, methodological variations exist across studies and highlight how variations in experimental protocols may contribute to differences observed across studies. Variations in neutrophil preparation (purified cells vs. whole blood) can significantly alter cellular responses, since purification eliminates influencing physiological factors, such as plasma proteins, cytokines, platelets, and other immune cells that normally modulate neutrophil activation. The choice of stimulus is also critical and using non-physiological stimuli such as PMA may not be clinically relevant. NETosis regulation in clinical settings is far more complex than the PMA-driven pathways typically modelled *in vitro*, and drug effect under simplified conditions may not directly translate to patient physiology. Prior literature has demonstrated that using patient plasma to stimulate NETosis provides a physiologically relevant environment through endogenous circulating mediators^[7]^. Furthermore, confounding drug effect (e.g., incubation times, prolonged handing, extended pre-incubation) may inadvertently activate or stress neutrophils. Collectively, these variations in methodological protocols highlight the complexity of modeling NETosis and underscore the need for cautious interpretations.

Lidocaine has received wide attention due its antitumor properties while simultaneously providing relief from cancer associated pain^[101–104]^. However, the exact mechanisms through which lidocaine exerts its antitumor effects remain largely unclear. In the context of perioperative NETs, patients with NSCLC who underwent VATS and were treated with lidocaine presented greater decreases in MPO, H3Cit and tumor metastasis biomarkers levels on postoperative day one than patients in the placebo group^[66]^. Similar findings regarding reduced NETs formation by lidocaine infusion were reported by Zhang and colleagues in the setting of pancreatectomy for malignancy. Nevertheless, lidocaine infusion did not lead to beneficial impact on overall or disease-free survival and NETs expression in tumor tissues^[105]^. The potential mechanism may be that lidocaine inhibits the activation of the HMGB-1 and granulocyte-colony stimulating factor (G-CSF). HMGB-1 and G-CSF significantly contribute to the stimulation of NETs release^[106]^. These findings highlight that anesthetic choice may extend beyond surgical recovery. However, further trials in perioperative cancer are still needed to validate current investigations.

### Opioids

The association between opioid use and alterations in the host immune system is well-established and has been recognized since the early 1800s. Opioids have varying effects on immune function and are influenced by factors such as the drug used, individual characteristics, and the time of exposure. Morphine, fentanyl, remifentanil, methadone, and codeine have considerable immunomodulatory effects, but tramadol, hydrocodone, oxycodone, and buprenorphine have considerably lower or no immune-modulating properties^[107]^. Opioids are known to modulate neutrophil function. They can inhibit bactericidal function through the inhibition of superoxide formation^[108–110]^. In addition, morphine has a suppressive effect on the recruitment and activation of neutrophils due to alterations in IL-8-induced neutrophil chemotaxis^[111,112]^. In a murine study, chronic morphine uses delayed wound healing due to a significant reduction in neutrophil and macrophage recruitment to the wound site. While a growing body of literature indicates that opioids are linked to immune response modulation, their specific impact on NETs formation remains unexplored. To date, no studies have been conducted to evaluate the impact of opioids on the NETs forming capacity of neutrophils. Given the various uses of opioids in clinical settings and the roles of NETs in many conditions, further preclinical and clinical studies are very much needed to elucidate our understanding of the roles of opioids in modulating NETosis. Table 2 outlines current knowledge gaps and proposes future directions for research in this area.

**Table 2:** Current knowledge gaps and future directions

Aspect	Current status	Identified gap	Future research need
Opioid impact on NETosis	No published data	NETs formation under opioid exposure not evaluated	Preclinical and clinical studies
Dexmedetomidine’s NETs mechanism	Hypothetical pathway suggested	Lack of direct mechanistic evidence	In vitro and translational studies
Comparative anesthetic studies	Few direct comparisons between anesthetics on NETs exists	Need for large, standardized comparisons	Multi-center randomized controlled trials
Dose–response relationships	Mixed evidence, especially in local anesthetics	Unclear thresholds for NETs inhibition	Pharmacodynamic and concentration-response investigations

NETs, neutrophil extracellular traps.

## Conclusion

Neutrophils and NETs play crucial roles in host defence, hemostasis and thrombosis, surgical trauma, and wound healing processes. As a result, they are significant in the fields of anesthesia and surgery. While several factors may play a role during the perioperative period, compelling evidence suggests that anesthetic drugs may have an impact on neutrophils and their ability to form NETs. The extent to which their influence significantly affects the short- and long-term outcomes of surgical patients has yet to be confirmed. In this review, we have compiled key evidence highlighting the impact of common anesthetics on NETs formation and their potential role in perioperative immune modulation. We emphasize the significant influence of anesthetics on the regulation of neutrophils, which may alter postoperative outcomes. A better understanding of the relationship between anesthetic exposure and NETosis could inform its implications in clinical practice and reveal new strategies to optimize perioperative care, particularly for immunocompromised patients. However, there is a lack of current evidence concerning the effects of anesthetics on NETs, especially their clinical implications in surgical and anesthetised patients. Preliminary evidence suggests that anesthetic agents can either suppress or have no effect on NETs formation, depending on the agent. However, the exact mechanisms by which different anesthetics influence NETs formation remain poorly understood, and significant gaps exist in mechanistic and translational studies. Future research should focus on exploring the mechanisms through which anesthetics regulate NETosis. In addition, future large-scale prospective trials are needed to compare the effects of different anesthetic regimens on the dynamics of NETs biomarkers and determine whether this will translate into clinically significant long-term outcomes and recovery, most importantly in cancer. These may enable effective perioperative strategies to optimize and improve clinical management of oncological-related surgeries.

## Data Availability

No new data were generated or analyzed in this review.
